# Crystal structure of potassium [4-amino-5-(benzo[*d*]thia­zol-2-yl)-6-(methyl­sulfan­yl)pyrimidin-2-yl](phenyl­sulfon­yl)aza­nide di­methyl­formamide monosolvate hemihydrate

**DOI:** 10.1107/S2056989019002275

**Published:** 2019-02-19

**Authors:** Rasha A. Azzam, Galal H. Elgemeie, Rokia R. Osman, Peter G. Jones

**Affiliations:** a Chemistry Department, Faculty of Science, Helwan University, Cairo, Egypt; b Institut für Anorganische und Analytische Chemie, Technische Universität Braunschweig, Hagenring 30, D-38106 Braunschweig, Germany

**Keywords:** crystal structure, benzo­thia­zole, sulfonamide, pyrimidine, hydrogen bonding

## Abstract

The title compound, K^+^·C_18_H_14_N_5_O_2_S_3_^−^·C_3_H_7_NO·0.5H_2_O, was obtained in a reaction designed to deliver a neutral 2-pyrimidylbenzo­thia­zole. The anion is deprotonated at the sulfonamide nitro­gen. The asymmetric unit of the title compound contains two potassium cations, two anions, two mol­ecules of DMF and one of water. The anions display some conformational differences but each contains an intra­molecular N—H⋯N_benzo­thia­zole_ hydrogen bond. The potassium ions both display a highly irregular six-coordination, different for each potassium ion. The anions, together with the DMF and water mol­ecules, are linked by four classical hydrogen bonds to form chains parallel to the *b-*axis direction.

## Abstract 2

The title compound, the potassium salt of a benzo­thia­zol(methyl­sulfan­yl)pyriminidine, was obtained in a reaction designed to deliver a neutral 2-pyrimidylbenzo­thia­zole. It crystallized with two independent mol­ecular units in the asymmetric unit.

## Chemical context   

**Figure 1:**
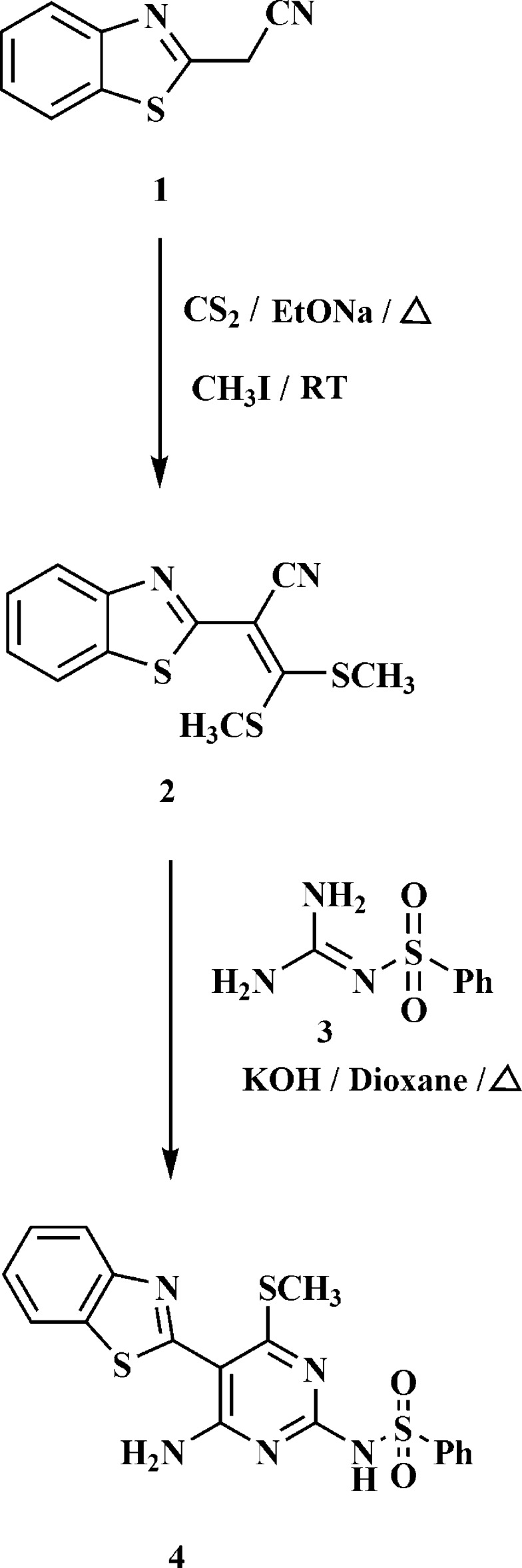
The attempted synthesis of compound **4**.

Benzo­thia­zoles are versatile heterocyclic biologically active compounds that are common in a variety of pharmaceutical preparations (Azzam *et al.*, 2017*a* ▸,*b* ▸). These compounds are of great importance in the field of medicinal chemistry because of their remarkable pharmacological potential (Keri *et al.*, 2015 ▸). Benzo­thia­zole derivatives show a high degree of structural diversity that has proved beneficial in the search for new therapeutic agents (Gill *et al.*, 2015 ▸). Research in benzo­thia­zole-based medicinal chemistry has rapidly become an active topic, since numerous benzo­thia­zole-based compounds have been widely used as clinical drugs to treat various types of diseases with high therapeutic potency (Sharma *et al.*, 2013 ▸). The medicinal properties associated with benzo­thia­zole-related drugs have encouraged medicinal chemists to synthesize a large number of new therapeutics (Elgemeie & Elghandour, 1990 ▸; Elgemeie *et al.*, 2000*a* ▸,*b* ▸). In recent years, 2-pyrimidylbenzo­thia­zoles have appeared as an important pharmacological class in the development of anti-tumor agents (Das *et al.*, 2003 ▸); their promising biological profile and synthetic accessibility have been attractive in their design and development as potential chemotherapeutics. In order to access this class of compounds in high yield and to introduce diversity, a variety of new synthetic methods has been invented (Seenaiah *et al.*, 2014 ▸). Recently, we have described the syntheses of various anti­metabolites starting from heterocyclic and acyclic cyano­ketene di­thio­acetals (Elgemeie *et al.*, 2015 ▸, 2016 ▸, 2017 ▸). As part of this program the reaction of 2-(benzo[*d*]thia­zol-2-yl)-3,3-bis­(methyl­thio)­acrylo­nitrile (**2**) with *N*-(di­amino­methyl­ene)benzene­sulfonamide (**3**) was studied (Fig. 1 ▸). The reaction between **2** and **3** in KOH/dioxane gave a product that was crystallized from DMF and identified by X-ray crystallography as the title compound, **5**, rather than the expected neutral 2-pyrimidylbenzo­thia­zole derivative, **4**. Compound **4** appears to be formed, at least in part, on dissolving **5** in deuterated DMSO; ^1^H NMR measurements showed the free NH proton at δ 11.50 ppm. However, we have still been unable to isolate and crystallize derivative **4**.
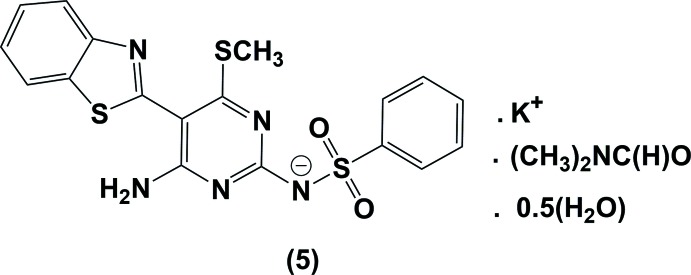


The formation of **5** from the reaction of **2** and **3** is assumed to proceed *via* initial addition of the amino group of **3** to the double bond of **2**, followed by elimination of CH_3_SH and cyclization *via* addition of the amino group to the cyano group of benzo­thia­zole to give the product **4**, which separated as its potassium salt **5** in the presence of KOH in the reaction medium. The ^1^H NMR spectra of the product **4**, formed in part in solution in deuterated DMSO, revealed the presence of a pyrimidine methyl­thio group at δ = 2.19 ppm and an amino group at δ = 8.49 ppm in solution. Compound **5** and its derivatives showed inter­esting preclinical anti­viral biological results compared to current anti­viral drugs and are currently being patented (Elgemeie *et al.*, 2018 ▸).

## Structural commentary   

**Figure 2:**
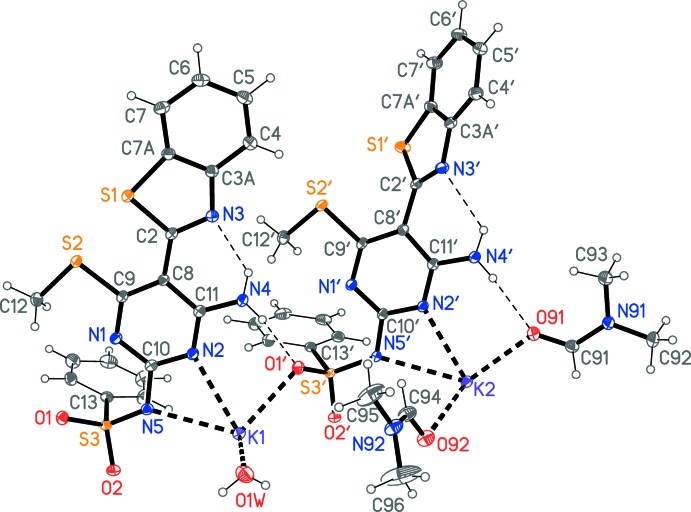
The mol­ecular structure of compound **5**, with the atom labelling (anion 1 has unprimed atom labels, anion 2 has primed atom labels). Displacement ellipsoids are drawn at the 50% probability level. Dashed lines indicate contacts to the potassium ions (thick) or classical hydrogen bonds (thin). For clarity, the sulfonamide phenyl group is not labelled.

The X-ray crystal structure indicated the exclusive presence of structure **5** in the solid state. The mol­ecular structure of compound **5** is illustrated in Fig. 2 ▸. The asymmetric unit contains two potassium cations, two anions of **4** deprotonated at the sulfonamide nitro­gen, two mol­ecules of DMF and one of water; it was chosen arbitrarily in an attempt to maximize the number of weak inter­actions (bonds to potassium, hydrogen bonds) within this unit.

**Figure 3:**
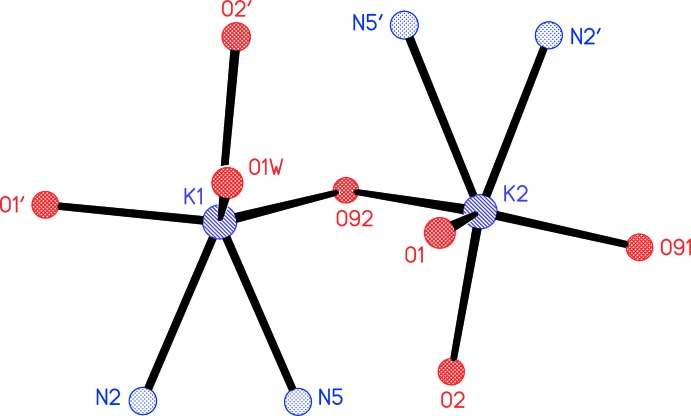
Coordination of the two potassium ions, showing the bridging nature of the DMF oxygen atom O92. Atoms O2′, O91, O92, K2, N2′, N5′ have been transformed to (−*x* + 1, −*y* + 1, −*z* + 1) and O1 to (−*x* + 1, −*y* + 2, −*z* + 1). The K1⋯K2 distance is 3.7975 (4) Å.

The potassium ions both display a highly irregular six-coordination; all K—N and K—O contacts (Table 1 ▸) are < 2.92 Å, and the next longest are > 3.33 Å. The atom K1 is coordinated by the pyrimidine nitro­gen atom N2 and the deprotonated sulfonamide nitro­gen N5, the sulfonamide oxygen atom O1′ and the water oxygen O1*W* within the asymmetric unit, and by the sulfonamide oxygen atom O2′ and the DMF oxygen O92 at (−*x* + 1, −*y* + 1, −*z* + 1). The atom K2 is coordinated by N2′, N5′ and both DMF oxygen atoms within the asymmetric unit, plus O2 at (−*x* + 1, −*y* + 1, −*z* + 1) and O1 at (*x*, *y* − 1, *z*). The angles subtended by the chelating anions *via* N2/N5 are particularly narrow. The bridging nature of O92 is shown in Fig. 3 ▸.

**Figure 4:**
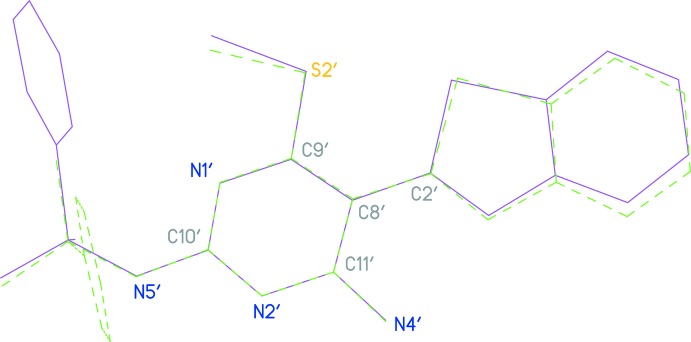
A view of the mol­ecular fit of the two independent anions of compound **5**. Fitted atoms are labelled; anion 1 (inverted from the refined coordinates) is green, anion 2 (primed atoms) is purple.

Each anion displays an intra­molecular hydrogen bond (N4—H01⋯N3 and N4′—H01′⋯N3′; Table 2 ▸), forming an *S*(6) ring motif. The anions display some differences in conformation; the angle between the benzo­thia­zole ring (seven atoms) and the pyrimidine ring plus immediate substituents (ten atoms) is 20.56 (5)° for anion 1 (unprimed atoms) but 42.20 (2)° for anion 2 (primed atoms). Comparing the torsion angles in Table 1 ▸, it may be seen that the signs of the torsion angles C9—C8—C2—S1 are different for the two anions [22.06 (18) and −42.51 (16)°]. A mol­ecular fit of the ten atoms of the pyrimidine ring, inverting one anion, gives an r.m.s. deviation of 0.03 Å (Fig. 4 ▸); the benzo­thia­zole rings are then a better fit, but the phenyl rings of the sulfonamide groups then point to opposite sides in the two anions, *cf*. torsion angle C10—N5—S3—C13 is 62.49 (11)°, and 65.28 (11)° in the non-inverted system.

## Supra­molecular features   

**Figure 5:**
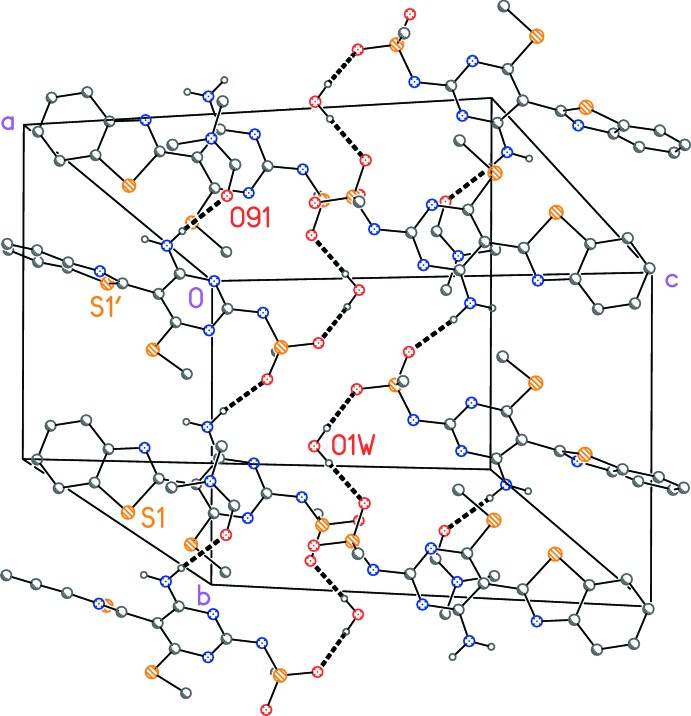
A view normal to plane (101) of the crystal packing of compound **5**. Hydrogen bonds (Table 2 ▸) are shown as dashed lines; hydrogen atoms not involved in hydrogen bonding have been omitted for clarity. Also omitted is the DMF mol­ecule based on atom O92 (which does not form any hydrogen bonds). For clarity, the phenyl ring at C13 is reduced to the *ipso* carbon.

Classical hydrogen bonds are shown in Table 2 ▸. These four hydrogen bonds combined with the contacts at the potassium ions give a highly complex packing pattern. If the potassium ions are omitted, a much more simple pattern emerges; the residues are linked *via* the water mol­ecules to form chains parallel to the *b-*axis direction, two of which are shown in Fig. 5 ▸.

## Database survey   

A search of the Cambridge Structural Database (CSD, V5.40, November 2018; Groom *et al.*, 2016 ▸) gave 47 hits (including ten duplicated structures) for the fragment consisting of a pyrimidine ring system bearing a two-coordinate, and thus negatively charged, nitro­gen substituent at the ring carbon between the two nitro­gen atoms, with the nitro­gen substituent forming part of a sulfonamide system. The hits included a silver salt (ASULDZ; Cook & Turner, 1975 ▸) and a sodium salt (JUBGUI/01; Hannan & Talukdar, 1992 ▸; Patel, 1995 ▸).

## Synthesis and crystallization   

**Synthesis of potassium [4-amino-5-(benzo[** ***d*** **]thia­zol-2-yl)-6-(methyl­sulfan­yl)pyrimidin-2-yl](phenyl­sulfon­yl)aza­nide di­meth­yl­formamide monosolvate hemihydrate (5):**

The reaction pathway is illustrated in Fig. 1 ▸. 2-(Benzo[*d*]thia­zole-2-yl)-3,3-bis­(methyl­thio)­acrylo­nitrile (**2**) (0.01 mol) was added to a stirred solution of the *N*-(di­amino­methyl­ene)benzene­sulfonamide (**3**) (0.01 mol) in dry dioxane (20 ml) containing potassium hydroxide (0.01 mol); the reaction mixture was refluxed for 2 h. After completion of the reaction (TLC), the solid precipitate was filtered off, and then recrystallized from DMF/H_2_O to give colourless block-like crystals of compound **5**, the potassium salt of compound **4**, in 75% yield (m.p. = 517 K). IR (KBr, cm^−1^): ν 3431 and 3874 (NH, NH_2_). ^1^H NMR (400 MHz, DMSO-d_6_): δ 2.19 (*s*, 3H, SCH_3_), 7.32–7.39 (*m*, 4H, 3CH-phenyl, CH benzo­thia­zole), 7.46 (*t*, 1H, *J* = 8.0 Hz, CH benzo­thia­zole), 7.83–7.85 (*m*, 2H, 2CH-phen­yl), 7.92 (*d*, 1H, *J* = 8.0 Hz, CH benzo­thia­zole), 8.01 (*d*, 1H, *J* = 8.0 Hz, CH benzo­thia­zole), 8.49 (*s*, *br*, 2H, NH_2_), 11.50 (*s*, *br*, 1H, NH).

## Refinement   

Crystal data, data collection and structure refinement details are summarized in Table 3 ▸. The NH and OH hydrogen atoms were identified in difference-Fourier maps and refined freely. Methyl groups were identified from difference-Fourier maps, idealized and refined as rigid groups [C—H = 0.98 Å, H—C—H = 109.5° with *U*_iso_(H) = 1.5*U*_eq_(C-meth­yl)], and allowed to rotate but not to tip (AFIX 137). Other hydrogen atoms were included using a riding model starting from calculated positions: C—H_aromatic_ = 0.95 Å with *U*_iso_(H) = 1.2*U*_eq_(C).

## Supplementary Material

Crystal structure: contains datablock(s) I, global. DOI: 10.1107/S2056989019002275/su5477sup1.cif

Structure factors: contains datablock(s) I. DOI: 10.1107/S2056989019002275/su5477Isup2.hkl

CCDC reference: 1896740

Additional supporting information:  crystallographic information; 3D view; checkCIF report

## Figures and Tables

**Table 1:** Selected geometric parameters (Å, °)

K1—N2	2.8371 (10)	K2—N2′	2.8320 (10)
K1—N5	2.9038 (11)	K2—N5′	2.9133 (10)
K1—O1′	2.6918 (9)	K2—O1^ii^	2.6846 (10)
K1—O2′^i^	2.7811 (9)	K2—O2^i^	2.6782 (9)
K1—O1*W*	2.8991 (13)	K2—O91	2.6594 (10)
K1—O92^i^	2.6779 (11)	K2—O92	2.8193 (10)
			
O92^i^—K1—O1′	122.10 (3)	O91—K2—O1^ii^	122.24 (3)
O92^i^—K1—O2′^i^	89.19 (3)	O2^i^—K2—O1^ii^	104.71 (3)
O1′—K1—O2′^i^	94.53 (3)	O91—K2—O92	116.05 (3)
O92^i^—K1—N2	117.13 (3)	O2^i^—K2—O92	74.59 (3)
O1′—K1—N2	72.98 (3)	O1^ii^—K2—O92	120.48 (3)
O2′^i^—K1—N2	153.65 (3)	O91—K2—N2′	78.44 (3)
O92^i^—K1—O1*W*	125.24 (3)	O2^i^—K2—N2′	145.92 (3)
O1′—K1—O1*W*	105.49 (3)	O1^ii^—K2—N2′	108.90 (3)
O2′^i^—K1—O1*W*	59.75 (3)	O92—K2—N2′	92.77 (3)
N2—K1—O1*W*	100.51 (3)	O91—K2—N5′	124.93 (3)
O92^i^—K1—N5	80.48 (3)	O2^i^—K2—N5′	145.82 (3)
O1′—K1—N5	116.98 (3)	O1^ii^—K2—N5′	83.45 (3)
O2′^i^—K1—N5	147.63 (3)	O92—K2—N5′	72.82 (3)
N2—K1—N5	46.67 (3)	N2′—K2—N5′	46.50 (3)
O1*W*—K1—N5	101.98 (3)	K1^i^—O92—K2	87.35 (3)
O91—K2—O2^i^	79.15 (3)		
			
S1—C2—C8—C9	22.06 (18)	S1′—C2′—C8′—C9′	−42.51 (16)
N1—C10—N5—S3	15.18 (17)	N1′—C10′—N5′—S3′	−15.33 (16)
C13—S3—N5—C10	62.49 (11)	C13′—S3′—N5′—C10′	65.28 (11)

Symmetry codes: (i) 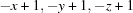; (ii) 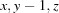.

**Table 2:** Hydrogen-bond geometry (Å, °)

*D*—H⋯*A*	*D*—H	H⋯*A*	*D*⋯*A*	*D*—H⋯*A*
N4—H01⋯N3	0.85 (2)	2.01 (2)	2.6859 (15)	136 (2)
N4′—H01′⋯N3′	0.87 (2)	2.26 (2)	2.8781 (15)	129 (2)
N4—H02⋯O1′	0.87 (2)	2.06 (2)	2.9205 (14)	168 (2)
N4′—H02′⋯O91	0.847 (19)	2.180 (19)	3.0207 (14)	172 (2)
O1*W*—H03⋯O2′^i^	0.81 (2)	2.07 (2)	2.8314 (15)	157 (2)
O1*W*—H04⋯O1^iii^	0.82 (3)	2.00 (3)	2.8211 (15)	176 (3)

Symmetry codes: (i) 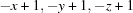; (iii) 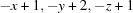.

**Table 3:** Experimental details

Crystal data	
Chemical formula	K^+^·C_18_H_14_N_5_O_2_S_3_^−^·C_3_H_7_NO·0.5H_2_O
*M*_r_	549.72
Crystal system, space group	Triclinic, *P* 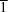
Temperature (K)	100
*a*, *b*, *c* (Å)	11.8407 (2), 12.6001 (4), 18.8671 (5)
α, β, γ (°)	90.160 (2), 102.361 (2), 117.933 (3)
*V* (Å^3^)	2411.88 (13)
*Z*	4
Radiation type	Mo *K*α
μ (mm^−1^)	0.52
Crystal size (mm)	0.35 × 0.25 × 0.25

Data collection	
Diffractometer	Oxford Diffraction Xcalibur Eos
Absorption correction	Multi-scan (*CrysAlis PRO*; Rigaku OD, 2015 ▸)
*T*_min_, *T*_max_	0.994, 1.000
No. of measured, independent and observed [*I* > 2σ(*I*)] reflections	256622, 13912, 11954
*R*_int_	0.047
(sin θ/λ)_max_ (Å^−1^)	0.704

Refinement	
*R*[*F* ^2^ > 2σ(*F* ^2^)], *wR*(*F* ^2^), *S*	0.030, 0.077, 1.05
No. of reflections	13912
No. of parameters	652
H-atom treatment	H atoms treated by a mixture of independent and constrained refinement
Δρ_max_, Δρ_min_ (e Å^−3^)	0.49, −0.40

Computer programs: *CrysAlis PRO* (Rigaku OD, 2015 ▸), *SHELXS97* (Sheldrick, 2008 ▸), *SHELXL2017* (Sheldrick, 2015 ▸) and *XP* (Siemens, 1994 ▸).

## supplementary crystallographic information

### Crystal data

**Table:**

K^+^·C_18_H_14_N_5_O_2_S_3_^−^·C_3_H_7_NO·0.5H_2_O	*Z* = 4
*M**_r_* = 549.72	*F*(000) = 1140
Triclinic, *P*1	*D*_x_ = 1.514 Mg m^−^^3^
*a* = 11.8407 (2) Å	Mo *K*α radiation, λ = 0.71073 Å
*b* = 12.6001 (4) Å	Cell parameters from 60104 reflections
*c* = 18.8671 (5) Å	θ = 2.4–30.6°
α = 90.160 (2)°	µ = 0.52 mm^−^^1^
β = 102.361 (2)°	*T* = 100 K
γ = 117.933 (3)°	Block, colourless
*V* = 2411.88 (13) Å^3^	0.35 × 0.25 × 0.25 mm

### Data collection

**Table:**

Oxford Diffraction Xcalibur Eos diffractometer	13912 independent reflections
Radiation source: fine-focus sealed X-ray tube	11954 reflections with *I* > 2σ(*I*)
Detector resolution: 16.1419 pixels mm^-1^	*R*_int_ = 0.047
ω–scan	θ_max_ = 30.0°, θ_min_ = 2.2°
Absorption correction: multi-scan (CrysAlis PRO; Rigaku OD, 2015)	*h* = −16→16
*T*_min_ = 0.994, *T*_max_ = 1.000	*k* = −17→17
256622 measured reflections	*l* = −26→26

### Refinement

**Table:**

Refinement on *F*^2^	Primary atom site location: structure-invariant direct methods
Least-squares matrix: full	Secondary atom site location: difference Fourier map
*R*[*F*^2^ > 2σ(*F*^2^)] = 0.030	Hydrogen site location: mixed
*wR*(*F*^2^) = 0.077	H atoms treated by a mixture of independent and constrained refinement
*S* = 1.05	*w* = 1/[σ^2^(*F*_o_^2^) + (0.0331*P*)^2^ + 1.360*P*] where *P* = (*F*_o_^2^ + 2*F*_c_^2^)/3
13912 reflections	(Δ/σ)_max_ = 0.002
652 parameters	Δρ_max_ = 0.49 e Å^−^^3^
0 restraints	Δρ_min_ = −0.40 e Å^−^^3^

### Special details

**Table:**

Geometry. All esds (except the esd in the dihedral angle between two l.s. planes) are estimated using the full covariance matrix. The cell esds are taken into account individually in the estimation of esds in distances, angles and torsion angles; correlations between esds in cell parameters are only used when they are defined by crystal symmetry. An approximate (isotropic) treatment of cell esds is used for estimating esds involving l.s. planes. Least-squares planes (x,y,z in crystal coordinates) and deviations from them (* indicates atom used to define plane) 7.5026 (0.0024) x + 0.9321 (0.0047) y + 8.4090 (0.0051) z = 6.8996 (0.0032) * -0.0075 (0.0009) C2 * 0.0127 (0.0009) N3 * 0.0119 (0.0012) C3A * -0.0103 (0.0011) C4 * -0.0129 (0.0011) C5 * 0.0036 (0.0011) C6 * 0.0057 (0.0010) C7 * 0.0136 (0.0012) C7A * -0.0168 (0.0007) S1 Rms deviation of fitted atoms = 0.0113 5.7089 (0.0026) x + 5.0746 (0.0027) y + 5.8404 (0.0040) z = 8.9926 (0.0015) Angle to previous plane (with approximate esd) = 20.556 ( 0.046 ) * 0.0683 (0.0011) C8 * 0.0132 (0.0010) C9 * 0.0105 (0.0010) C10 * -0.0228 (0.0011) C11 * -0.0190 (0.0009) N1 * -0.0234 (0.0010) N2 * 0.1204 (0.0009) C2 * -0.1062 (0.0006) S2 * 0.0646 (0.0008) N5 * -0.1055 (0.0009) N4 -0.1967 (0.0016) C12 -0.2593 (0.0011) S3 Rms deviation of fitted atoms = 0.0689=============================================================================== - 2.2187 (0.0034) x + 11.9323 (0.0012) y - 3.9110 (0.0057) z = 2.1027 (0.0024) * 0.0421 (0.0009) C2' * -0.0030 (0.0009) N3' * -0.0322 (0.0011) C3A' * -0.0032 (0.0011) C4' * 0.0107 (0.0011) C5' * 0.0163 (0.0011) C6' * 0.0101 (0.0010) C7' * -0.0308 (0.0011) C7A' * -0.0099 (0.0007) S1' Rms deviation of fitted atoms = 0.0220 4.0185 (0.0027) x + 7.7469 (0.0023) y + 2.9794 (0.0039) z = 6.0513 (0.0008) Angle to previous plane (with approximate esd) = 42.200 ( 0.021 ) * -0.0154 (0.0011) C8' * 0.0164 (0.0010) C9' * 0.0066 (0.0010) C10' * 0.0299 (0.0010) C11' * 0.0483 (0.0009) N1' * 0.0253 (0.0009) N2' * -0.1309 (0.0009) C2' * 0.0515 (0.0006) S2' * -0.1005 (0.0008) N5' * 0.0688 (0.0008) N4' -0.2532 (0.0017) C12' 0.1802 (0.0010) S3' Rms deviation of fitted atoms = 0.0625===============================================================================

### Fractional atomic coordinates and isotropic or equivalent isotropic displacement parameters (Å^2^)

**Table:**

	*x*	*y*	*z*	*U*_iso_*/*U*_eq_	
S1	0.67977 (4)	1.02828 (3)	0.09802 (2)	0.01751 (7)	
S2	0.47162 (3)	1.04245 (3)	0.15477 (2)	0.01496 (6)	
S3	0.31676 (3)	0.90940 (3)	0.39554 (2)	0.01143 (6)	
C2	0.63692 (12)	0.90552 (11)	0.15096 (6)	0.0128 (2)	
C3A	0.73099 (13)	0.85659 (11)	0.07477 (7)	0.0142 (2)	
C4	0.77389 (14)	0.78717 (12)	0.04155 (7)	0.0191 (3)	
H4	0.762989	0.712529	0.057998	0.023*	
C5	0.83241 (14)	0.82984 (13)	−0.01570 (7)	0.0202 (3)	
H5	0.862215	0.784025	−0.038767	0.024*	
C6	0.84838 (14)	0.93991 (13)	−0.04020 (7)	0.0198 (3)	
H6	0.889986	0.967721	−0.079226	0.024*	
C7	0.80507 (14)	1.00885 (12)	−0.00895 (7)	0.0189 (3)	
H7	0.814762	1.082683	−0.026224	0.023*	
C7A	0.74648 (13)	0.96563 (11)	0.04907 (7)	0.0149 (2)	
C8	0.57484 (12)	0.89569 (10)	0.21128 (6)	0.0121 (2)	
C9	0.50259 (12)	0.95499 (10)	0.22093 (6)	0.0121 (2)	
C10	0.46477 (12)	0.87455 (11)	0.32734 (6)	0.0123 (2)	
C11	0.57609 (12)	0.81497 (11)	0.26460 (6)	0.0126 (2)	
C12	0.37416 (14)	1.08905 (12)	0.19405 (7)	0.0172 (2)	
H12A	0.417727	1.120507	0.245597	0.026*	
H12B	0.286843	1.019598	0.190301	0.026*	
H12C	0.364720	1.152476	0.167707	0.026*	
C13	0.17010 (12)	0.82029 (11)	0.32687 (6)	0.0134 (2)	
C14	0.11572 (15)	0.69479 (12)	0.32221 (8)	0.0217 (3)	
H14	0.159533	0.658842	0.352897	0.026*	
C15	−0.00368 (15)	0.62278 (13)	0.27195 (8)	0.0260 (3)	
H15	−0.041467	0.537174	0.268056	0.031*	
C16	−0.06766 (14)	0.67590 (14)	0.22751 (8)	0.0232 (3)	
H16	−0.149850	0.626424	0.193940	0.028*	
C17	−0.01210 (14)	0.80064 (13)	0.23196 (8)	0.0219 (3)	
H17	−0.055603	0.836490	0.200925	0.026*	
C18	0.10750 (13)	0.87374 (12)	0.28186 (7)	0.0172 (2)	
H18	0.145806	0.959352	0.285031	0.021*	
N1	0.44791 (11)	0.94544 (9)	0.27717 (6)	0.0132 (2)	
N2	0.52377 (11)	0.80718 (9)	0.32239 (6)	0.0135 (2)	
N3	0.67040 (11)	0.82571 (10)	0.13235 (6)	0.0148 (2)	
N4	0.63003 (12)	0.74264 (10)	0.26054 (6)	0.0176 (2)	
H02	0.6135 (19)	0.6870 (18)	0.2900 (11)	0.033 (5)*	
H01	0.651 (2)	0.7365 (18)	0.2208 (11)	0.034 (5)*	
N5	0.41916 (11)	0.86521 (10)	0.38931 (6)	0.0137 (2)	
O1	0.35584 (9)	1.03538 (8)	0.38428 (5)	0.01534 (17)	
O2	0.28269 (9)	0.87920 (8)	0.46485 (5)	0.01566 (18)	
S1'	0.61437 (3)	0.29511 (3)	0.01676 (2)	0.01725 (7)	
S2'	0.54376 (3)	0.46946 (3)	0.09428 (2)	0.01721 (7)	
S3'	0.46192 (3)	0.42692 (3)	0.35846 (2)	0.01176 (6)	
C2'	0.72463 (12)	0.34847 (11)	0.10370 (6)	0.0122 (2)	
C3A'	0.85618 (13)	0.34467 (11)	0.03647 (7)	0.0145 (2)	
C4'	0.97234 (14)	0.36291 (13)	0.01881 (7)	0.0193 (3)	
H4'	1.051620	0.390641	0.055865	0.023*	
C5'	0.97023 (15)	0.34001 (13)	−0.05340 (8)	0.0209 (3)	
H5'	1.048490	0.351042	−0.065639	0.025*	
C6'	0.85444 (15)	0.30083 (12)	−0.10871 (7)	0.0209 (3)	
H6'	0.855166	0.285520	−0.157894	0.025*	
C7'	0.73941 (15)	0.28421 (12)	−0.09258 (7)	0.0198 (3)	
H7'	0.661289	0.259304	−0.130140	0.024*	
C7A'	0.74080 (13)	0.30496 (11)	−0.01957 (7)	0.0150 (2)	
C8'	0.68045 (12)	0.36129 (10)	0.16870 (6)	0.0115 (2)	
C9'	0.59510 (12)	0.40880 (10)	0.17095 (6)	0.0120 (2)	
C10'	0.58805 (12)	0.36622 (10)	0.28789 (6)	0.0114 (2)	
C11'	0.72036 (12)	0.32086 (10)	0.23520 (6)	0.0117 (2)	
C12'	0.41439 (15)	0.48795 (13)	0.11841 (7)	0.0206 (3)	
H12D	0.348305	0.410299	0.129017	0.031*	
H12E	0.372818	0.515917	0.077628	0.031*	
H12F	0.451606	0.547696	0.161728	0.031*	
C13'	0.31457 (13)	0.37337 (12)	0.28815 (6)	0.0144 (2)	
C14'	0.24643 (13)	0.25133 (12)	0.26169 (7)	0.0163 (2)	
H14'	0.280816	0.199034	0.279043	0.020*	
C15'	0.12741 (14)	0.20642 (13)	0.20955 (7)	0.0205 (3)	
H15'	0.081078	0.123450	0.190146	0.025*	
C16'	0.07620 (15)	0.28272 (15)	0.18583 (7)	0.0234 (3)	
H16'	−0.006040	0.251404	0.150880	0.028*	
C17'	0.14437 (15)	0.40440 (15)	0.21284 (8)	0.0242 (3)	
H17'	0.108590	0.456059	0.196406	0.029*	
C18'	0.26492 (15)	0.45107 (13)	0.26390 (7)	0.0199 (3)	
H18'	0.312684	0.534651	0.281964	0.024*	
N1'	0.55003 (11)	0.41385 (9)	0.22932 (5)	0.01266 (19)	
N2'	0.67301 (10)	0.32232 (9)	0.29374 (5)	0.01215 (19)	
N3'	0.84368 (11)	0.36751 (10)	0.10580 (6)	0.0146 (2)	
N4'	0.80664 (11)	0.27784 (10)	0.24374 (6)	0.0154 (2)	
H02'	0.8068 (18)	0.2361 (17)	0.2788 (10)	0.026 (5)*	
H01'	0.8381 (19)	0.2724 (17)	0.2073 (11)	0.032 (5)*	
N5'	0.53703 (11)	0.35582 (9)	0.34781 (5)	0.01244 (19)	
O1'	0.53653 (10)	0.55695 (8)	0.35774 (5)	0.01774 (19)	
O2'	0.41688 (10)	0.39421 (8)	0.42515 (5)	0.01618 (18)	
O1W	0.74945 (12)	0.84359 (11)	0.54810 (6)	0.0277 (2)	
H03	0.721 (2)	0.779 (2)	0.5641 (12)	0.043 (6)*	
H04	0.722 (3)	0.881 (2)	0.5691 (14)	0.059 (7)*	
K1	0.49406 (3)	0.68951 (3)	0.45040 (2)	0.01683 (6)	
K2	0.61675 (3)	0.18387 (2)	0.41184 (2)	0.01349 (6)	
C91	0.80325 (13)	0.04442 (12)	0.39544 (7)	0.0162 (2)	
H91	0.767210	0.011982	0.435682	0.019*	
C92	0.90128 (15)	−0.08901 (13)	0.40894 (8)	0.0220 (3)	
H92A	0.859007	−0.110674	0.449887	0.033*	
H92B	0.996418	−0.057876	0.427120	0.033*	
H92C	0.864741	−0.160831	0.373445	0.033*	
C93	0.93416 (16)	0.04719 (14)	0.31230 (8)	0.0245 (3)	
H93A	0.914558	0.111216	0.294568	0.037*	
H93B	0.896673	−0.019652	0.273044	0.037*	
H93C	1.029863	0.079505	0.327664	0.037*	
N91	0.87702 (12)	0.00353 (10)	0.37388 (6)	0.0171 (2)	
O91	0.77808 (10)	0.12169 (9)	0.36692 (5)	0.01859 (19)	
C94	0.80107 (14)	0.47812 (13)	0.48372 (8)	0.0227 (3)	
H94	0.776435	0.467817	0.431813	0.027*	
C95	0.96954 (19)	0.68087 (16)	0.47527 (10)	0.0424 (5)	
H95A	0.931121	0.654844	0.422790	0.064*	
H95B	1.062670	0.701335	0.486721	0.064*	
H95C	0.962128	0.752100	0.488784	0.064*	
C96	0.9427 (3)	0.60669 (19)	0.59444 (10)	0.0733 (9)	
H96A	1.030708	0.614032	0.610117	0.110*	
H96B	0.880906	0.539661	0.615825	0.110*	
H96C	0.945776	0.682111	0.610803	0.110*	
N92	0.89949 (12)	0.58360 (10)	0.51602 (6)	0.0225 (2)	
O92	0.73809 (10)	0.39159 (9)	0.51491 (5)	0.0222 (2)	

### Atomic displacement parameters (Å^2^)

**Table:**

	*U*^11^	*U*^22^	*U*^33^	*U*^12^	*U*^13^	*U*^23^
S1	0.02517 (18)	0.01570 (14)	0.01810 (14)	0.01219 (13)	0.01195 (12)	0.00747 (11)
S2	0.01847 (16)	0.01588 (14)	0.01479 (13)	0.01093 (12)	0.00581 (11)	0.00621 (11)
S3	0.01119 (14)	0.01133 (12)	0.01105 (12)	0.00464 (11)	0.00318 (10)	0.00208 (9)
C2	0.0127 (6)	0.0121 (5)	0.0126 (5)	0.0052 (5)	0.0028 (4)	0.0032 (4)
C3A	0.0134 (6)	0.0159 (5)	0.0133 (5)	0.0071 (5)	0.0031 (4)	0.0019 (4)
C4	0.0214 (7)	0.0201 (6)	0.0193 (6)	0.0118 (5)	0.0074 (5)	0.0033 (5)
C5	0.0201 (7)	0.0250 (7)	0.0181 (6)	0.0121 (6)	0.0065 (5)	0.0004 (5)
C6	0.0167 (7)	0.0280 (7)	0.0141 (6)	0.0096 (6)	0.0050 (5)	0.0032 (5)
C7	0.0198 (7)	0.0219 (6)	0.0156 (6)	0.0095 (5)	0.0065 (5)	0.0057 (5)
C7A	0.0155 (6)	0.0171 (6)	0.0133 (5)	0.0083 (5)	0.0046 (4)	0.0026 (4)
C8	0.0133 (6)	0.0112 (5)	0.0120 (5)	0.0055 (5)	0.0041 (4)	0.0030 (4)
C9	0.0115 (6)	0.0110 (5)	0.0126 (5)	0.0047 (4)	0.0023 (4)	0.0029 (4)
C10	0.0101 (6)	0.0125 (5)	0.0123 (5)	0.0040 (5)	0.0023 (4)	0.0024 (4)
C11	0.0114 (6)	0.0118 (5)	0.0129 (5)	0.0047 (5)	0.0019 (4)	0.0024 (4)
C12	0.0183 (7)	0.0189 (6)	0.0180 (6)	0.0120 (5)	0.0038 (5)	0.0028 (5)
C13	0.0117 (6)	0.0146 (5)	0.0124 (5)	0.0048 (5)	0.0040 (4)	0.0013 (4)
C14	0.0213 (7)	0.0154 (6)	0.0223 (6)	0.0057 (5)	0.0015 (5)	0.0037 (5)
C15	0.0230 (8)	0.0161 (6)	0.0260 (7)	0.0008 (6)	0.0017 (6)	0.0003 (5)
C16	0.0143 (7)	0.0268 (7)	0.0191 (6)	0.0036 (6)	0.0009 (5)	−0.0039 (5)
C17	0.0184 (7)	0.0274 (7)	0.0200 (6)	0.0130 (6)	−0.0002 (5)	−0.0008 (5)
C18	0.0165 (7)	0.0176 (6)	0.0184 (6)	0.0095 (5)	0.0030 (5)	0.0005 (5)
N1	0.0135 (5)	0.0141 (5)	0.0130 (5)	0.0072 (4)	0.0040 (4)	0.0038 (4)
N2	0.0143 (5)	0.0143 (5)	0.0137 (5)	0.0076 (4)	0.0047 (4)	0.0039 (4)
N3	0.0169 (6)	0.0157 (5)	0.0143 (5)	0.0088 (4)	0.0061 (4)	0.0036 (4)
N4	0.0261 (7)	0.0183 (5)	0.0174 (5)	0.0154 (5)	0.0109 (5)	0.0087 (4)
N5	0.0133 (5)	0.0166 (5)	0.0131 (5)	0.0082 (4)	0.0046 (4)	0.0041 (4)
O1	0.0151 (5)	0.0109 (4)	0.0176 (4)	0.0043 (3)	0.0039 (3)	0.0015 (3)
O2	0.0170 (5)	0.0183 (4)	0.0124 (4)	0.0081 (4)	0.0059 (3)	0.0033 (3)
S1'	0.01307 (16)	0.02409 (16)	0.01137 (13)	0.00726 (13)	0.00081 (11)	−0.00157 (11)
S2'	0.02224 (17)	0.02347 (15)	0.01330 (14)	0.01611 (14)	0.00615 (12)	0.00867 (11)
S3'	0.01608 (15)	0.01188 (13)	0.01004 (12)	0.00854 (11)	0.00399 (10)	0.00284 (9)
C2'	0.0135 (6)	0.0116 (5)	0.0099 (5)	0.0053 (5)	0.0016 (4)	0.0022 (4)
C3A'	0.0174 (6)	0.0149 (5)	0.0126 (5)	0.0084 (5)	0.0047 (5)	0.0037 (4)
C4'	0.0193 (7)	0.0250 (6)	0.0171 (6)	0.0127 (6)	0.0060 (5)	0.0054 (5)
C5'	0.0249 (8)	0.0252 (7)	0.0207 (6)	0.0156 (6)	0.0122 (5)	0.0075 (5)
C6'	0.0290 (8)	0.0218 (6)	0.0150 (6)	0.0131 (6)	0.0093 (5)	0.0030 (5)
C7'	0.0224 (7)	0.0214 (6)	0.0128 (6)	0.0088 (6)	0.0031 (5)	−0.0011 (5)
C7A'	0.0165 (6)	0.0141 (5)	0.0139 (5)	0.0065 (5)	0.0045 (5)	0.0009 (4)
C8'	0.0105 (6)	0.0116 (5)	0.0108 (5)	0.0042 (4)	0.0020 (4)	0.0021 (4)
C9'	0.0119 (6)	0.0099 (5)	0.0121 (5)	0.0042 (4)	0.0016 (4)	0.0030 (4)
C10'	0.0113 (6)	0.0096 (5)	0.0118 (5)	0.0043 (4)	0.0014 (4)	0.0013 (4)
C11'	0.0102 (6)	0.0112 (5)	0.0118 (5)	0.0041 (4)	0.0011 (4)	0.0008 (4)
C12'	0.0236 (7)	0.0284 (7)	0.0179 (6)	0.0194 (6)	0.0041 (5)	0.0058 (5)
C13'	0.0161 (6)	0.0213 (6)	0.0110 (5)	0.0123 (5)	0.0053 (4)	0.0039 (4)
C14'	0.0164 (7)	0.0211 (6)	0.0141 (5)	0.0102 (5)	0.0058 (5)	0.0030 (5)
C15'	0.0165 (7)	0.0272 (7)	0.0152 (6)	0.0075 (6)	0.0064 (5)	0.0014 (5)
C16'	0.0161 (7)	0.0421 (8)	0.0150 (6)	0.0159 (6)	0.0048 (5)	0.0061 (5)
C17'	0.0267 (8)	0.0400 (8)	0.0186 (6)	0.0256 (7)	0.0069 (5)	0.0092 (6)
C18'	0.0254 (8)	0.0259 (7)	0.0161 (6)	0.0185 (6)	0.0055 (5)	0.0043 (5)
N1'	0.0136 (5)	0.0137 (5)	0.0117 (4)	0.0074 (4)	0.0031 (4)	0.0035 (4)
N2'	0.0122 (5)	0.0135 (5)	0.0112 (4)	0.0069 (4)	0.0019 (4)	0.0022 (4)
N3'	0.0145 (5)	0.0179 (5)	0.0118 (5)	0.0080 (4)	0.0039 (4)	0.0034 (4)
N4'	0.0169 (6)	0.0217 (5)	0.0126 (5)	0.0131 (5)	0.0042 (4)	0.0048 (4)
N5'	0.0154 (5)	0.0140 (5)	0.0114 (4)	0.0097 (4)	0.0035 (4)	0.0035 (4)
O1'	0.0255 (5)	0.0119 (4)	0.0171 (4)	0.0094 (4)	0.0065 (4)	0.0032 (3)
O2'	0.0212 (5)	0.0196 (4)	0.0117 (4)	0.0118 (4)	0.0067 (3)	0.0040 (3)
O1W	0.0339 (7)	0.0209 (5)	0.0306 (6)	0.0110 (5)	0.0177 (5)	0.0049 (4)
K1	0.02118 (15)	0.01917 (13)	0.01416 (12)	0.01203 (11)	0.00636 (10)	0.00500 (9)
K2	0.01341 (13)	0.01430 (12)	0.01346 (11)	0.00694 (10)	0.00394 (9)	0.00372 (9)
C91	0.0155 (6)	0.0176 (6)	0.0155 (5)	0.0076 (5)	0.0047 (5)	0.0019 (4)
C92	0.0273 (8)	0.0219 (6)	0.0239 (7)	0.0167 (6)	0.0081 (6)	0.0079 (5)
C93	0.0295 (8)	0.0312 (7)	0.0257 (7)	0.0208 (7)	0.0165 (6)	0.0128 (6)
N91	0.0193 (6)	0.0188 (5)	0.0182 (5)	0.0118 (5)	0.0077 (4)	0.0064 (4)
O91	0.0206 (5)	0.0208 (5)	0.0190 (4)	0.0130 (4)	0.0062 (4)	0.0039 (4)
C94	0.0196 (7)	0.0230 (7)	0.0179 (6)	0.0054 (6)	0.0012 (5)	0.0036 (5)
C95	0.0359 (10)	0.0305 (9)	0.0324 (9)	−0.0041 (8)	0.0014 (7)	0.0163 (7)
C96	0.097 (2)	0.0295 (9)	0.0224 (9)	−0.0215 (11)	0.0030 (10)	−0.0015 (7)
N92	0.0224 (6)	0.0163 (5)	0.0193 (5)	0.0029 (5)	0.0023 (5)	0.0050 (4)
O92	0.0209 (5)	0.0163 (4)	0.0219 (5)	0.0036 (4)	0.0037 (4)	0.0033 (4)

### Geometric parameters (Å, º)

**Table:**

S1—C7A	1.7299 (13)	S3'—N5'	1.5672 (10)
S1—C2	1.7716 (12)	S3'—C13'	1.7778 (13)
S2—C9	1.7600 (12)	C2'—N3'	1.3058 (17)
S2—C12	1.7957 (13)	C2'—C8'	1.4685 (16)
S3—O2	1.4500 (9)	C3A'—N3'	1.3915 (15)
S3—O1	1.4611 (9)	C3A'—C4'	1.3986 (19)
S3—N5	1.5770 (11)	C3A'—C7A'	1.4046 (18)
S3—C13	1.7767 (13)	C4'—C5'	1.3843 (18)
C2—N3	1.3126 (16)	C4'—H4'	0.9500
C2—C8	1.4575 (16)	C5'—C6'	1.401 (2)
C3A—N3	1.3859 (16)	C5'—H5'	0.9500
C3A—C7A	1.4008 (17)	C6'—C7'	1.379 (2)
C3A—C4	1.4020 (18)	C6'—H6'	0.9500
C4—C5	1.3822 (19)	C7'—C7A'	1.3964 (17)
C4—H4	0.9500	C7'—H7'	0.9500
C5—C6	1.403 (2)	C8'—C9'	1.4024 (17)
C5—H5	0.9500	C8'—C11'	1.4256 (16)
C6—C7	1.3818 (19)	C9'—N1'	1.3360 (15)
C6—H6	0.9500	C10'—N2'	1.3432 (15)
C7—C7A	1.3966 (17)	C10'—N1'	1.3519 (15)
C7—H7	0.9500	C10'—N5'	1.3708 (15)
C8—C9	1.4100 (17)	C11'—N2'	1.3451 (15)
C8—C11	1.4354 (16)	C11'—N4'	1.3462 (16)
C9—N1	1.3346 (15)	C12'—H12D	0.9800
C10—N2	1.3419 (16)	C12'—H12E	0.9800
C10—N1	1.3507 (15)	C12'—H12F	0.9800
C10—N5	1.3726 (15)	C13'—C14'	1.3877 (18)
C11—N4	1.3454 (16)	C13'—C18'	1.3929 (17)
C11—N2	1.3474 (15)	C14'—C15'	1.3893 (19)
C12—H12A	0.9800	C14'—H14'	0.9500
C12—H12B	0.9800	C15'—C16'	1.387 (2)
C12—H12C	0.9800	C15'—H15'	0.9500
C13—C18	1.3877 (17)	C16'—C17'	1.386 (2)
C13—C14	1.3943 (18)	C16'—H16'	0.9500
C14—C15	1.393 (2)	C17'—C18'	1.390 (2)
C14—H14	0.9500	C17'—H17'	0.9500
C15—C16	1.390 (2)	C18'—H18'	0.9500
C15—H15	0.9500	N4'—H02'	0.847 (19)
C16—C17	1.385 (2)	N4'—H01'	0.87 (2)
C16—H16	0.9500	O1W—H03	0.81 (2)
C17—C18	1.3938 (19)	O1W—H04	0.82 (3)
C17—H17	0.9500	C91—O91	1.2380 (15)
C18—H18	0.9500	C91—N91	1.3291 (17)
K1—N2	2.8371 (10)	C91—H91	0.9500
K1—N5	2.9038 (11)	C92—N91	1.4548 (16)
K1—O1'	2.6918 (9)	C92—H92A	0.9800
K1—O2'^i^	2.7811 (9)	C92—H92B	0.9800
K1—O1W	2.8991 (13)	C92—H92C	0.9800
K1—O92^i^	2.6779 (11)	C93—N91	1.4497 (17)
K2—N2'	2.8320 (10)	C93—H93A	0.9800
K2—N5'	2.9133 (10)	C93—H93B	0.9800
K2—O1^ii^	2.6846 (10)	C93—H93C	0.9800
K2—O2^i^	2.6782 (9)	C94—O92	1.2333 (17)
K2—O91	2.6594 (10)	C94—N92	1.3177 (18)
K2—O92	2.8193 (10)	C94—H94	0.9500
N4—H02	0.87 (2)	C95—N92	1.4516 (19)
N4—H01	0.85 (2)	C95—H95A	0.9800
S1'—C7A'	1.7299 (13)	C95—H95B	0.9800
S1'—C2'	1.7612 (12)	C95—H95C	0.9800
S2'—C9'	1.7639 (12)	C96—N92	1.439 (2)
S2'—C12'	1.8002 (14)	C96—H96A	0.9800
S3'—O1'	1.4541 (9)	C96—H96B	0.9800
S3'—O2'	1.4566 (9)	C96—H96C	0.9800
			
C7A—S1—C2	89.78 (6)	N2'—C11'—N4'	115.39 (10)
C9—S2—C12	100.78 (6)	N2'—C11'—C8'	121.93 (11)
O2—S3—O1	113.35 (5)	N4'—C11'—C8'	122.68 (11)
O2—S3—N5	106.98 (5)	S2'—C12'—H12D	109.5
O1—S3—N5	116.05 (6)	S2'—C12'—H12E	109.5
O2—S3—C13	106.02 (6)	H12D—C12'—H12E	109.5
O1—S3—C13	106.14 (6)	S2'—C12'—H12F	109.5
N5—S3—C13	107.73 (6)	H12D—C12'—H12F	109.5
N3—C2—C8	122.89 (11)	H12E—C12'—H12F	109.5
N3—C2—S1	113.58 (9)	C14'—C13'—C18'	121.11 (12)
C8—C2—S1	123.50 (9)	C14'—C13'—S3'	118.40 (9)
N3—C3A—C7A	114.97 (11)	C18'—C13'—S3'	120.36 (10)
N3—C3A—C4	125.16 (11)	C13'—C14'—C15'	119.30 (12)
C7A—C3A—C4	119.86 (12)	C13'—C14'—H14'	120.3
C5—C4—C3A	118.46 (12)	C15'—C14'—H14'	120.3
C5—C4—H4	120.8	C16'—C15'—C14'	120.01 (13)
C3A—C4—H4	120.8	C16'—C15'—H15'	120.0
C4—C5—C6	120.89 (13)	C14'—C15'—H15'	120.0
C4—C5—H5	119.6	C17'—C16'—C15'	120.38 (13)
C6—C5—H5	119.6	C17'—C16'—H16'	119.8
C7—C6—C5	121.59 (12)	C15'—C16'—H16'	119.8
C7—C6—H6	119.2	C16'—C17'—C18'	120.21 (13)
C5—C6—H6	119.2	C16'—C17'—H17'	119.9
C6—C7—C7A	117.32 (12)	C18'—C17'—H17'	119.9
C6—C7—H7	121.3	C17'—C18'—C13'	118.96 (13)
C7A—C7—H7	121.3	C17'—C18'—H18'	120.5
C7—C7A—C3A	121.87 (12)	C13'—C18'—H18'	120.5
C7—C7A—S1	128.54 (10)	C9'—N1'—C10'	116.17 (10)
C3A—C7A—S1	109.57 (9)	C10'—N2'—C11'	117.31 (10)
C9—C8—C11	113.91 (11)	C10'—N2'—K2	101.07 (7)
C9—C8—C2	125.26 (10)	C11'—N2'—K2	136.87 (8)
C11—C8—C2	120.71 (11)	C2'—N3'—C3A'	111.15 (10)
N1—C9—C8	124.03 (11)	C11'—N4'—H02'	116.1 (13)
N1—C9—S2	115.19 (9)	C11'—N4'—H01'	119.3 (13)
C8—C9—S2	120.71 (9)	H02'—N4'—H01'	121.2 (18)
N2—C10—N1	125.23 (11)	C10'—N5'—S3'	120.32 (8)
N2—C10—N5	113.88 (10)	C10'—N5'—K2	96.61 (7)
N1—C10—N5	120.89 (11)	S3'—N5'—K2	143.06 (5)
N4—C11—N2	115.53 (11)	S3'—O1'—K1	114.51 (5)
N4—C11—C8	122.42 (11)	K1—O1W—H03	74.2 (16)
N2—C11—C8	122.05 (11)	K1—O1W—H04	93.9 (18)
S2—C12—H12A	109.5	H03—O1W—H04	103 (2)
S2—C12—H12B	109.5	O92^i^—K1—O1'	122.10 (3)
H12A—C12—H12B	109.5	O92^i^—K1—O2'^i^	89.19 (3)
S2—C12—H12C	109.5	O1'—K1—O2'^i^	94.53 (3)
H12A—C12—H12C	109.5	O92^i^—K1—N2	117.13 (3)
H12B—C12—H12C	109.5	O1'—K1—N2	72.98 (3)
C18—C13—C14	120.90 (12)	O2'^i^—K1—N2	153.65 (3)
C18—C13—S3	120.94 (10)	O92^i^—K1—O1W	125.24 (3)
C14—C13—S3	118.07 (10)	O1'—K1—O1W	105.49 (3)
C15—C14—C13	119.14 (13)	O2'^i^—K1—O1W	59.75 (3)
C15—C14—H14	120.4	N2—K1—O1W	100.51 (3)
C13—C14—H14	120.4	O92^i^—K1—N5	80.48 (3)
C16—C15—C14	120.15 (13)	O1'—K1—N5	116.98 (3)
C16—C15—H15	119.9	O2'^i^—K1—N5	147.63 (3)
C14—C15—H15	119.9	N2—K1—N5	46.67 (3)
C17—C16—C15	120.28 (13)	O1W—K1—N5	101.98 (3)
C17—C16—H16	119.9	O91—K2—O2^i^	79.15 (3)
C15—C16—H16	119.9	O91—K2—O1^ii^	122.24 (3)
C16—C17—C18	120.15 (13)	O2^i^—K2—O1^ii^	104.71 (3)
C16—C17—H17	119.9	O91—K2—O92	116.05 (3)
C18—C17—H17	119.9	O2^i^—K2—O92	74.59 (3)
C13—C18—C17	119.37 (12)	O1^ii^—K2—O92	120.48 (3)
C13—C18—H18	120.3	O91—K2—N2'	78.44 (3)
C17—C18—H18	120.3	O2^i^—K2—N2'	145.92 (3)
C9—N1—C10	116.76 (10)	O1^ii^—K2—N2'	108.90 (3)
C10—N2—C11	117.63 (10)	O92—K2—N2'	92.77 (3)
C10—N2—K1	101.67 (7)	O91—K2—N5'	124.93 (3)
C11—N2—K1	140.70 (8)	O2^i^—K2—N5'	145.82 (3)
C2—N3—C3A	112.08 (10)	O1^ii^—K2—N5'	83.45 (3)
C11—N4—H02	116.3 (13)	O92—K2—N5'	72.82 (3)
C11—N4—H01	116.8 (13)	N2'—K2—N5'	46.50 (3)
H02—N4—H01	122.6 (18)	O91—C91—N91	124.77 (12)
C10—N5—S3	121.03 (9)	O91—C91—H91	117.6
C10—N5—K1	97.74 (7)	N91—C91—H91	117.6
S3—N5—K1	137.67 (6)	N91—C92—H92A	109.5
S3—O1—K2^iii^	115.26 (5)	N91—C92—H92B	109.5
S3—O2—K2^i^	136.13 (5)	H92A—C92—H92B	109.5
C7A'—S1'—C2'	89.38 (6)	N91—C92—H92C	109.5
C9'—S2'—C12'	101.82 (6)	H92A—C92—H92C	109.5
O1'—S3'—O2'	113.04 (5)	H92B—C92—H92C	109.5
O1'—S3'—N5'	114.63 (6)	N91—C93—H93A	109.5
O2'—S3'—N5'	107.06 (5)	N91—C93—H93B	109.5
O1'—S3'—C13'	107.07 (6)	H93A—C93—H93B	109.5
O2'—S3'—C13'	104.46 (6)	N91—C93—H93C	109.5
N5'—S3'—C13'	110.11 (6)	H93A—C93—H93C	109.5
N3'—C2'—C8'	123.71 (11)	H93B—C93—H93C	109.5
N3'—C2'—S1'	114.91 (9)	C91—N91—C93	121.04 (11)
C8'—C2'—S1'	121.23 (9)	C91—N91—C92	122.18 (11)
N3'—C3A'—C4'	125.82 (12)	C93—N91—C92	116.75 (11)
N3'—C3A'—C7A'	114.92 (11)	C91—O91—K2	120.87 (8)
C4'—C3A'—C7A'	119.19 (11)	O92—C94—N92	125.77 (13)
C5'—C4'—C3A'	119.11 (13)	O92—C94—K2	52.85 (7)
C5'—C4'—H4'	120.4	N92—C94—K2	163.54 (11)
C3A'—C4'—H4'	120.4	O92—C94—H94	117.1
C4'—C5'—C6'	121.01 (13)	N92—C94—H94	117.1
C4'—C5'—H5'	119.5	K2—C94—H94	67.1
C6'—C5'—H5'	119.5	N92—C95—H95A	109.5
C7'—C6'—C5'	120.77 (12)	N92—C95—H95B	109.5
C7'—C6'—H6'	119.6	H95A—C95—H95B	109.5
C5'—C6'—H6'	119.6	N92—C95—H95C	109.5
C6'—C7'—C7A'	118.25 (13)	H95A—C95—H95C	109.5
C6'—C7'—H7'	120.9	H95B—C95—H95C	109.5
C7A'—C7'—H7'	120.9	N92—C96—H96A	109.5
C7'—C7A'—C3A'	121.65 (12)	N92—C96—H96B	109.5
C7'—C7A'—S1'	128.70 (11)	H96A—C96—H96B	109.5
C3A'—C7A'—S1'	109.58 (9)	N92—C96—H96C	109.5
C9'—C8'—C11'	114.77 (11)	H96A—C96—H96C	109.5
C9'—C8'—C2'	124.58 (10)	H96B—C96—H96C	109.5
C11'—C8'—C2'	120.62 (11)	C94—N92—C96	120.61 (13)
N1'—C9'—C8'	123.92 (11)	C94—N92—C95	122.44 (13)
N1'—C9'—S2'	116.05 (9)	C96—N92—C95	116.95 (14)
C8'—C9'—S2'	120.02 (9)	C94—O92—K1^i^	132.77 (10)
N2'—C10'—N1'	125.70 (11)	C94—O92—K2	106.74 (9)
N2'—C10'—N5'	113.44 (10)	K1^i^—O92—K2	87.35 (3)
N1'—C10'—N5'	120.83 (11)		
			
C7A—S1—C2—N3	−0.17 (11)	C3A'—C4'—C5'—C6'	1.0 (2)
C7A—S1—C2—C8	177.68 (11)	C4'—C5'—C6'—C7'	0.1 (2)
N3—C3A—C4—C5	179.77 (13)	C5'—C6'—C7'—C7A'	−1.4 (2)
C7A—C3A—C4—C5	0.8 (2)	C6'—C7'—C7A'—C3A'	1.59 (19)
C3A—C4—C5—C6	−0.1 (2)	C6'—C7'—C7A'—S1'	178.38 (11)
C4—C5—C6—C7	−0.8 (2)	N3'—C3A'—C7A'—C7'	176.71 (12)
C5—C6—C7—C7A	1.1 (2)	C4'—C3A'—C7A'—C7'	−0.55 (19)
C6—C7—C7A—C3A	−0.3 (2)	N3'—C3A'—C7A'—S1'	−0.63 (14)
C6—C7—C7A—S1	−178.52 (11)	C4'—C3A'—C7A'—S1'	−177.89 (10)
N3—C3A—C7A—C7	−179.64 (12)	C2'—S1'—C7A'—C7'	−175.59 (13)
C4—C3A—C7A—C7	−0.6 (2)	C2'—S1'—C7A'—C3A'	1.51 (9)
N3—C3A—C7A—S1	−1.16 (14)	N3'—C2'—C8'—C9'	142.01 (13)
C4—C3A—C7A—S1	177.90 (11)	S1'—C2'—C8'—C9'	−42.51 (16)
C2—S1—C7A—C7	179.08 (13)	N3'—C2'—C8'—C11'	−40.08 (17)
C2—S1—C7A—C3A	0.73 (10)	S1'—C2'—C8'—C11'	135.40 (10)
N3—C2—C8—C9	−160.28 (13)	C11'—C8'—C9'—N1'	−1.83 (17)
S1—C2—C8—C9	22.06 (18)	C2'—C8'—C9'—N1'	176.19 (11)
N3—C2—C8—C11	15.40 (19)	C11'—C8'—C9'—S2'	176.82 (9)
S1—C2—C8—C11	−162.25 (10)	C2'—C8'—C9'—S2'	−5.15 (17)
C11—C8—C9—N1	4.82 (18)	C12'—S2'—C9'—N1'	−12.29 (11)
C2—C8—C9—N1	−179.23 (12)	C12'—S2'—C9'—C8'	168.96 (10)
C11—C8—C9—S2	−171.83 (9)	C9'—C8'—C11'—N2'	3.63 (17)
C2—C8—C9—S2	4.12 (18)	C2'—C8'—C11'—N2'	−174.47 (11)
C12—S2—C9—N1	1.16 (11)	C9'—C8'—C11'—N4'	−176.93 (11)
C12—S2—C9—C8	178.09 (10)	C2'—C8'—C11'—N4'	4.96 (18)
C9—C8—C11—N4	174.05 (12)	O1'—S3'—C13'—C14'	159.94 (10)
C2—C8—C11—N4	−2.09 (19)	O2'—S3'—C13'—C14'	−79.93 (11)
C9—C8—C11—N2	−6.32 (17)	N5'—S3'—C13'—C14'	34.72 (12)
C2—C8—C11—N2	177.53 (11)	O1'—S3'—C13'—C18'	−24.15 (12)
O2—S3—C13—C18	113.30 (11)	O2'—S3'—C13'—C18'	95.97 (11)
O1—S3—C13—C18	−7.52 (12)	N5'—S3'—C13'—C18'	−149.37 (10)
N5—S3—C13—C18	−132.44 (11)	C18'—C13'—C14'—C15'	0.69 (19)
O2—S3—C13—C14	−63.18 (12)	S3'—C13'—C14'—C15'	176.57 (10)
O1—S3—C13—C14	175.99 (10)	C13'—C14'—C15'—C16'	−1.62 (19)
N5—S3—C13—C14	51.07 (12)	C14'—C15'—C16'—C17'	1.2 (2)
C18—C13—C14—C15	−0.6 (2)	C15'—C16'—C17'—C18'	0.1 (2)
S3—C13—C14—C15	175.93 (11)	C16'—C17'—C18'—C13'	−1.0 (2)
C13—C14—C15—C16	−0.4 (2)	C14'—C13'—C18'—C17'	0.6 (2)
C14—C15—C16—C17	1.1 (2)	S3'—C13'—C18'—C17'	−175.16 (10)
C15—C16—C17—C18	−1.0 (2)	C8'—C9'—N1'—C10'	−2.00 (18)
C14—C13—C18—C17	0.7 (2)	S2'—C9'—N1'—C10'	179.30 (9)
S3—C13—C18—C17	−175.65 (10)	N2'—C10'—N1'—C9'	4.65 (18)
C16—C17—C18—C13	0.0 (2)	N5'—C10'—N1'—C9'	−173.44 (11)
C8—C9—N1—C10	0.33 (18)	N1'—C10'—N2'—C11'	−2.95 (18)
S2—C9—N1—C10	177.15 (9)	N5'—C10'—N2'—C11'	175.27 (10)
N2—C10—N1—C9	−4.84 (18)	N1'—C10'—N2'—K2	−162.67 (10)
N5—C10—N1—C9	175.93 (11)	N5'—C10'—N2'—K2	15.55 (11)
N1—C10—N2—C11	3.35 (19)	N4'—C11'—N2'—C10'	179.06 (11)
N5—C10—N2—C11	−177.38 (11)	C8'—C11'—N2'—C10'	−1.46 (17)
N1—C10—N2—K1	−177.35 (11)	N4'—C11'—N2'—K2	−30.78 (17)
N5—C10—N2—K1	1.93 (12)	C8'—C11'—N2'—K2	148.69 (9)
N4—C11—N2—C10	−177.73 (11)	C8'—C2'—N3'—C3A'	178.01 (11)
C8—C11—N2—C10	2.63 (18)	S1'—C2'—N3'—C3A'	2.26 (13)
N4—C11—N2—K1	3.35 (19)	C4'—C3A'—N3'—C2'	176.01 (12)
C8—C11—N2—K1	−176.30 (9)	C7A'—C3A'—N3'—C2'	−1.04 (15)
C8—C2—N3—C3A	−178.31 (11)	N2'—C10'—N5'—S3'	166.36 (9)
S1—C2—N3—C3A	−0.45 (14)	N1'—C10'—N5'—S3'	−15.33 (16)
C7A—C3A—N3—C2	1.06 (16)	N2'—C10'—N5'—K2	−14.92 (10)
C4—C3A—N3—C2	−177.94 (13)	N1'—C10'—N5'—K2	163.39 (10)
N2—C10—N5—S3	−164.13 (9)	O1'—S3'—N5'—C10'	−55.50 (11)
N1—C10—N5—S3	15.18 (17)	O2'—S3'—N5'—C10'	178.27 (9)
N2—C10—N5—K1	−1.86 (11)	C13'—S3'—N5'—C10'	65.28 (11)
N1—C10—N5—K1	177.45 (10)	O1'—S3'—N5'—K2	126.62 (9)
O2—S3—N5—C10	176.11 (10)	O2'—S3'—N5'—K2	0.38 (11)
O1—S3—N5—C10	−56.26 (12)	C13'—S3'—N5'—K2	−112.60 (9)
C13—S3—N5—C10	62.49 (11)	O2'—S3'—O1'—K1	−20.89 (7)
O2—S3—N5—K1	22.74 (10)	N5'—S3'—O1'—K1	−143.97 (5)
O1—S3—N5—K1	150.37 (7)	C13'—S3'—O1'—K1	93.58 (6)
C13—S3—N5—K1	−90.88 (9)	O91—C91—N91—C93	−0.8 (2)
O2—S3—O1—K2^iii^	99.65 (6)	O91—C91—N91—C92	−178.96 (13)
N5—S3—O1—K2^iii^	−24.76 (7)	N91—C91—O91—K2	176.30 (10)
C13—S3—O1—K2^iii^	−144.38 (5)	O92—C94—N92—C96	−0.2 (3)
O1—S3—O2—K2^i^	−113.35 (8)	K2—C94—N92—C96	−79.3 (4)
N5—S3—O2—K2^i^	15.85 (9)	O92—C94—N92—C95	179.47 (17)
C13—S3—O2—K2^i^	130.61 (7)	K2—C94—N92—C95	100.3 (4)
C7A'—S1'—C2'—N3'	−2.26 (10)	N92—C94—O92—K1^i^	98.16 (17)
C7A'—S1'—C2'—C8'	−178.12 (10)	K2—C94—O92—K1^i^	−102.27 (11)
N3'—C3A'—C4'—C5'	−177.66 (12)	N92—C94—O92—K2	−159.57 (14)
C7A'—C3A'—C4'—C5'	−0.73 (19)		

Symmetry codes: (i) −*x*+1, −*y*+1, −*z*+1; (ii) *x*, *y*−1, *z*; (iii) *x*, *y*+1, *z*.

### Hydrogen-bond geometry (Å, º)

**Table:**

*D*—H···*A*	*D*—H	H···*A*	*D*···*A*	*D*—H···*A*
N4—H01···N3	0.85 (2)	2.01 (2)	2.6859 (15)	136.0 (18)
N4′—H01′···N3′	0.87 (2)	2.26 (2)	2.8781 (15)	128.5 (16)
N4—H02···O1′	0.87 (2)	2.06 (2)	2.9205 (14)	167.5 (18)
N4′—H02′···O91	0.847 (19)	2.180 (19)	3.0207 (14)	172.0 (17)
O1*W*—H03···O2′^i^	0.81 (2)	2.07 (2)	2.8314 (15)	157 (2)
O1*W*—H04···O1^iv^	0.82 (3)	2.00 (3)	2.8211 (15)	176 (3)

Symmetry codes: (i) −*x*+1, −*y*+1, −*z*+1; (iv) −*x*+1, −*y*+2, −*z*+1.
